# Acute bacterial lymphadenitis in children: a retrospective, cross-sectional study

**DOI:** 10.1007/s00431-023-04861-0

**Published:** 2023-03-07

**Authors:** Annaleise R. Howard-Jones, Khalfan Al Abdali, Philip N. Britton

**Affiliations:** 1 Centre for Infectious Diseases & Microbiology Laboratory Services, NSW Health Pathology – Institute of Clinical Pathology & Medical Research, Westmead Hospital, Westmead, New South Wales Australia; 2 Discipline of Child & Adolescent Health, Faculty of Medicine and Health, University of Sydney, New South Wales, Australia; 3 Department of Pediatrics, Pediatric Infectious Diseases Unit, Nizwa Hospital, Ministry of Health, Nizwa, Sultanate of Oman; 4 Department of Infectious Diseases & Microbiology, The Children’s Hospital at Westmead, Westmead, NSW Australia

**Keywords:** Bacterial lymphadenitis, Antimicrobial therapy, *Staphylococcus aureus*, Group A *Streptococcus*

## Abstract

Acute bacterial lymphadenitis is a common childhood condition, yet there remains considerable variability in antibiotic treatment choice, particularly in settings with low prevalence of methicillin-resistant *Staphylococcus aureus* such as Europe and Australasia. This retrospective cross-sectional study reviewed children presenting with acute bacterial lymphadenitis to a tertiary paediatric hospital in Australia between 1 October 2018 and 30 September 2020. Treatment approaches were analysed with respect to children with complicated versus uncomplicated disease. A total of 148 children were included in the study, encompassing 25 patients with complicated disease and 123 with uncomplicated lymphadenitis, as defined by the presence or absence of an associated abscess or collection. In culture-positive cases, methicillin-susceptible *S. aureus* (49%) and Group A Streptococcus (43%) predominated, while methicillin-resistant *S. aureus* was seen in a minority of cases (6%). Children with complicated disease generally presented later and had a prolonged length of stay, longer durations of antibiotics, and higher frequency of surgical intervention. Beta-lactam therapy (predominantly flucloxacillin or first-generation cephalosporins) formed the mainstay of therapy for uncomplicated disease, while treatment of complicated disease was more variable with higher rates of clindamycin use.

*    Conclusion*: Uncomplicated lymphadenitis can be managed with narrow-spectrum beta-lactam therapy (such as flucloxacillin) with low rates of relapse or complications. In complicated disease, early imaging, prompt surgical intervention, and infectious diseases consultation are recommended to guide antibiotic therapy. Prospective randomised trials are needed to guide optimal antibiotic choice and duration in children presenting with acute bacterial lymphadenitis, particularly in association with abscess formation, and to promote uniformity in treatment approaches.

**Table:** 

**What is Known:** *• Acute bacterial lymphadenitis is a common childhood infection.* *• Antibiotic prescribing practices are highly variable in bacterial lymphadenitis.*	
**What is New:** *• Uncomplicated bacterial lymphadenitis in children can be managed with single agent narrow-spectrum beta-lactam therapy in low-MRSA prevalence settings.* *• Further trials are needed to ascertain optimal treatment duration and the role of clindamycin in complicated disease.*

**Supplementary Information:**

The online version contains supplementary material available at 10.1007/s00431-023-04861-0.

## Introduction

Lymphadenopathy is a common presentation in childhood occurring in 44% of children between the ages of 0 and 5 years [1]. Acute lymphadenitis (with symptoms of up to 2 weeks of duration) has a broad differential diagnosis including pyogenic bacterial infections, poly- or mono-microbial anaerobic infections in association with dental or periodontal disease, *Bartonella henselae* and Toxoplasmosis as well as viral infections such as Epstein-Barr virus and cytomegalovirus [2]. Tuberculosis and non-tuberculous mycobacteria may also present as acute lymphadenopathy but more commonly present with a subacute or chronic course. Virulent, pyogenic skin commensals— *Staphylococcus aureus* and beta-haemolytic *Streptococci* such as Group A Streptococcus or (in neonates) Group B Streptococcus—predominate [3]. Contiguous foci of infection such as a retropharyngeal abscess may occur and should be considered in a child with suggestive clinical features. Bacteraemia is infrequent [3].

Given the high variability in methicillin-resistant *S. aureus* (MRSA) prevalence rates by geography, an understanding of local resistance patterns is important to guide empiric therapy. In settings with high prevalence rates of MRSA, such as the USA, this pathogen must necessarily be considered in empiric treatment regimens for acute lymphadenitis [3, 4]. In the USA, clindamycin is a recommended first-line antibiotic for acute pyogenic lymphadenitis [3, 4]. The degree to which these US-based regimens apply in low MRSA prevalence countries is unclear. In Australia, much of Europe and East Asia, 80–90% of *S. aureus* isolates are methicillin susceptible [5, 6], enabling a narrower spectrum in the empiric antimicrobial regimen for *S. aureus*-associated disease presentations. In a recent study from France, Group A Streptococcus (40%) and MSSA (23%) were the primary pathogens seen in a cohort of children with deep neck abscesses [7]. Similarly, a recent Korean study of cervical lymphadenitis revealed MSSA in 60% of cultured aspirates, Group A Streptococcus in 6% and 34% was sterile; none of the cultures grew MRSA [8]. In low-MRSA-prevalence regions such as Australia and Europe, narrow spectrum beta-lactam antibiotics (namely flucloxacillin or first-generation cephalosporins) are recommended as first-line therapy [9] due to the strong bactericidal activity of beta-lactams against MSSA and Group A Streptococcus, which remain the most prevalent pathogens.

This study aimed to describe the clinical features and microbiology of acute bacterial lymphadenitis in children as well as to analyse the management and outcomes of complicated versus uncomplicated disease in a low-MRSA-prevalence setting. The results of this retrospective cohort study may inform future prospective trials to more definitively answer the question of the optimal antibiotic regimen for acute bacterial lymphadenitis in children in low-MRSA-prevalence populations such as Australia, Europe, and East Asia.

## Methodologies

### Study design

This retrospective cross-sectional study was conducted at a tertiary children**’**s hospital in Sydney, Australia, and included children admitted to the hospital from the community or seen in the emergency department between 1 October 2018 and 30 September 2020. All patients with international classification of disease (ICD-10 Australian Modification)-coded discharge data with the terms “acute lymphadenitis (L04)”, “non-specific lymphadenitis (I88)”, or “enlarged lymph nodes (R59)” were identified retrospectively from the electronic medical record, with additional capture through a consultation log maintained by the infectious diseases team. Patients with diagnosed non-tuberculous mycobacterial lymphadenitis, tuberculous lymphadenitis, *Bartonella henselae* infection, Toxoplasmosis, mumps, cytomegalovirus or Epstein-Barr virus-associated lymphadenitis, Kawasaki disease, malignancy or other non-infectious lymphadenopathy, or those with symptoms of over 2 weeks of duration at time of presentation were excluded. Immunosuppressed patients were excluded including those with primary immunodeficiency, human immunodeficiency virus, malignancy, any past history of solid organ or bone marrow transplant or those receiving ≥ 1 mg/kg prednisone per day (or equivalent) for more than 1 week. Patients were excluded if they had been hospital inpatients at any time in the 30 days prior to initial presentation. Patients were also excluded if an alternative diagnosis was reached on subsequent imaging or biopsy results.

Participant groups were analysed and compared based on disease complexity. Patients with an abscess or collection associated with the region of lymphadenopathy (as determined by imaging, operative, or clinical findings during admission) were considered to have complicated disease and, the remaining patients, uncomplicated disease.

### Data collection

For all participants who met inclusion criteria, detailed demographic, clinical, laboratory, and radiological data were collected from the electronic medical record. Nationality and Aboriginal and Torres Strait Islander background was obtained from the medical records, derived by self-identification at time of registration. All data were de-identified prior to analysis.

### Statistical analysis

Categorical variables were represented as count and proportion, and continuous variables were represented as mean and standard deviation unless otherwise stated. Student**’**s independent t-test was used to compare differences between patient groups. A *p*-value of < 0.05 was regarded as statistically significant. Statistical analysis was performed using Microsoft Excel (Microsoft 365, version 2108) and Jamovi software (v 2.2.3) [10].

## Results

A total of 148 children with acute lymphadenitis were included in this study. Mean age at presentation was 4.4 ± 3.6 years with a slight male preponderance (male:female ratio of 1.18:1). Most children presenting were of Australian nationality (91.2%) with a small proportion self-identifying with Aboriginal and Torres Strait Islander heritage (2.7% of the total cohort). Most children presented with localised swelling (138 participants, 96.5%), predominantly involving the cervical area (131 participants, 94.9%) and mostly unilateral (122 participants, 87.8%), and fever (128 participants, 88.3%) (Table 1). Systemic symptoms were common, including lethargy/malaise (40 participants, 47.1%), vomiting (22 participants, 18.6%), and less frequently nausea and night sweats (5 participants, 5.6% each) (Table 1). Children with lymphadenitis presented after 4.2 ± 3.1 days of symptoms. All children were previously well with no medical comorbidities except for one child in the uncomplicated group with a known chromosomal deletion (data not shown).

**Table 1:** Demographics and clinical features in children presenting with lymphadenitis, stratified by severity

		**All**	**Uncomplicated disease**	**Complicated disease**	*p*
		*n* = 148	*n* = 123	*n* = 25	
Age at presentation, median (IQR)		3.7 (4.8)	4.0 (5.0)	3.0 (5.0)	0.381
Male: Female ratio		1.18: 1	1.16: 1	1.27: 1	0.832
Aboriginal or Torres Strait Islander background (%) ^a^		4 (2.7)	1 (0.8)	3 (12.0)	**0.002**
Country of birth ^a^					
Australia		135	113 (91.9)	22 (88)	0.536
New Zealand		3	2 (1.6)	1 (4)	
USA		2	2 (1.6)	0 (0)	
South Africa		2	1 (0.8)	1 (4)	
Syria		1	1 (0.8)	0 (0)	
China		1	1 (0.8)	0 (0)	
Croatia		1	1 (0.8)	0 (0)	
Nepal		1	1 (0.8)	0 (0)	
UK		1	1 (0.8)	0 (0)	
Lebanon		1	0 (0)	1 (4)	
**Days from symptom onset to presentation, mean (SD)**		4.2 (3.1)	3.8 (2.8)	6.0 (3.8)	**0.001**
**Presenting features**					
Fever		88.3%	87.6%	91.7%	0.575
Mass/swelling		96.5%	97.5%	91.3%	0.140
Unilateral		87.8%	83.9%	90.5%	0.718
Cervical		94.9%	94.9%	95.3%	0.738
Inguinal		5.1%	5.1%	4.8%	0.957
Axillary		1.4%	1.7%	0.0%	0.553
Lethargy/malaise		47.1%	45.2%	58.3%	0.405
Nausea		5.6%	6.4%	0.0%	0.393
Vomiting		18.6%	17.8%	23.5%	0.580
Night sweats		5.6%	6.3%	0.0%	0.396

^a^ Country of birth and identification with Aboriginal and Torres Strait Islander background was obtained from the medical records, derived by self-identification at time of registration

Of the 148 cases, 25 (16.9%) were categorised as having complicated disease and 123 (83.1%) uncomplicated disease. The children with complicated disease presented later on average than those with uncomplicated lymphadenitis (6.0 ± 3.8 versus 3.8 ± 2.8 days, *p* = 0.001), and children identifying as having Aboriginal or Torres Strait Islander background were significantly over-represented in this group (12.0% versus 0.8%, *p* = 0.002) (Table 1). Other demographic characteristics and presenting features were not significantly different between the two groups.

Not unexpectedly, laboratory markers of inflammation at presentation were more elevated in the complicated disease group with C-reactive protein significantly higher (104.8 ± 71.9 versus 66.2 ± 64.3, *p* = 0.009); although white cell count and neutrophil count were numerically higher in the complicated disease group, the difference did not reach statistical significance. In patients for whom inflammatory markers were repeated on days 3–7, neutrophil count was significantly higher in the complicated disease group (10.8 ± 4.2 versus 6.5 ± 4.8, *p* = 0.034), though average counts in both groups were lower compared to the time of presentation. There was no significant difference in the organisms identified from blood culture, intra-operative culture, or carriage swabs in the complicated versus uncomplicated groups. In line with case definitions, a collection or abscess on imaging was seen significantly more often in the complicated disease group (95.8% versus 0%, *p* < 0.001) as was infectious arteritis (4.2% versus 0.0%, *p* = 0.041), while lymphadenitis without a necrotic centre (4.2% versus 29.0%, *p* = 0.010) and lymphadenitis not specified (8.3% versus 46.0%, *p* < 0.001) were seen significantly less frequently in the complicated disease group (Table 2). In line with the more complex disease and later presentation, patients in the complicated group were significantly more likely to have a computed tomography (CT) scan (45.8% versus 2.0%, *p* < 0.001) and less likely to undergo ultrasonography (54.2% versus 94.0%, *p* < 0.001) compared to the uncomplicated group.

**Table 2:** Laboratory and radiological findings in children presenting with lymphadenitis

		**Uncomplicated disease**	**Complicated disease**	*p*
		*n* = 123	*n* = 25	
**Laboratory findings at presentation**				
White cell count, mean (SD)		18.3 (8.2)	20.7 (6.6)	0.171
Neutrophils, mean (SD)		12.1 (7.4)	14.3 (6.9)	0.197
C-reactive protein, mean (SD)		66.2 (64.3)	104.8 (71.9)	**0.009**
**Laboratory findings at days 3–7 (maximum values)**				
White cell count, mean (SD)		13.7 (6.6)	14.3 (5.4)	0.828
Neutrophils, mean (SD)		6.5 (4.8)	10.8 (4.2)	**0.034**
C-reactive protein, mean (SD)		43.3 (36.4)	46.8 (36.1)	0.820
Acute kidney injury, *n* (%)		0/116 (0.0)	1/20 (5.0)	**0.015**
**Culture results**				
Blood cultures – *n* (%)		1/115 (0.9)	1/23 (4.3)	0.231
Organisms:				
Methicillin-susceptible *Staphylococcus aureus*		0/93 (0.0)	1/23 (4.3)	-
Group B Streptococcus		1/115 (0.9)	0/23 (0.0)	-
Intra-operative cultures (tissue/pus/swabs)—*n* (%)				
Organisms:				
Methicillin-susceptible *Staphylococcus aureus*		7/11 (63.6)	8/12 (66.7)	0.886
Methicillin-resistant *Staphylococcus aureus*		1/11 (9.1)	0/12 (0.0)	0.307
Group A Streptococcus		3/11 (27.3)	4/12 (33.3)	0.765
Cultures for carriage (nose/throat swab)—*n* (%)				
Organisms:		2/11 (18.2)	2/3 (66.7)	0.115
Methicillin-susceptible *Staphylococcus aureus*				
Methicillin-resistant *Staphylococcus aureus*		1/11 (9.1)	0/1 (0.0)	0.689
Group A Streptococcus		8/11 (72.7)	1/3 (33.3)	0.561
**Imaging findings**				
Imaging performed (Y/N), *n* (%)		100/123 (81.3)	24/25 (96.0)	0.070
Ultrasound		94/100 (94.0)	13/24 (54.2)	**< 0.001**
Computed tomography		2/100 (2.0)	11/24 (45.8)	**< 0.001**
Magnetic resonance imaging		1/100 (1.0)	0/24 (0)	0.626
X-ray (lateral airway)		3/100 (3.0)	0/24 (0)	0.394
Day of imaging, from presentation, mean (SD)		0.8 (1.8)	1.3 (1.4)	0.238
Findings on imaging, *n* (%):				
Normal findings		3/100 (3.0)	0/24 (0)	0.394
Lymphadenitis with necrotic centre		22/100 (22.0)	1/24 (4.2)	**0.044**
Lymphadenitis without necrotic centre		29/100 (29.0)	1/24 (4.2)	**0.010**
Lymphadenitis not specified		46/100 (46.0)	3/24 (12.5)	**0.002**
Para- or retro-pharyngeal collection or abscess		0/100 (0.0)	23/24 (95.8)	**< 0.001**
Other (infectious arteritis)		0/100 (0.0)	1/24 (4.2)	**0.041**

In total (including all patients with positive blood cultures, intra-operative samples (tissue, pus, or swabs), and carriage swabs), MSSA was isolated from 17 patients (48.6%), Group A Streptococcus from 15 patients (42.9%), MRSA from only 2 patients (5.7%), and Group B Streptococcus from 1 patient (2.9%). Only two children were bacteraemic (1.4% of total cohort), one 18-day-old neonate with Group B Streptococcus and one 9-month-old infant with MSSA bacteraemia. Operative sterile site specimens were positive in 23 cases, including 15 with MSSA (65.2%), seven with Group A Streptococcus isolates (30.4%) and only one with MRSA (4.3%).

Overall, complication rates were low and relapse uncommon (4%). The children in the complicated disease group had a more severe disease course than the uncomplicated group. Their length of stay was significantly longer (5.5 ± 2.7 versus 3.5 ± 2.1 days, *p* < 0.001) as well as the total duration of antibiotics (13.5 ± 5.8 versus 9.7 ± 3.4 days, *p* < 0.001), and also the duration of both IV and oral antibiotics individually (Table 3). Surgery was performed in over half of the children with complicated disease, which represented a significant difference from the uncomplicated group (56.0% versus 8.1%, *p* < 0.001). In culture positive cases, the pathogen identified did not seem to influence the frequency of surgical intervention or duration of antibiotic therapy (Supplementary Table 2). The rate of infectious disease consultation was numerically higher in the complicated disease group, but the difference did not reach statistical significance (16.0% versus 8.9%, *p* = 0.290). The time to antibiotic initiation, time to defervescence, and adverse event rates were not significantly different between the two groups. No children required intensive care unit admission, and relapse rates were low in both groups (4.0% versus 4.1%, *p* = 0.988).

**Table 3:** Clinical parameters and outcomes for children with lymphadenitis, stratified by severity

	**Uncomplicated disease**	**Complicated disease**	*p*
	*n* = 123	*n* = 25	
Days from admission to defervescence, mean (SD)	1.6 (1.2)	1.9 (1.7)	0.383
Length of stay (days), mean (SD)	3.5 (2.1)	5.5 (2.7)	**< 0.001 **^**a**^
Duration of antibiotics used, mean (SD)			
Total duration antibiotics	9.7 (3.4)	13.5 (5.8)	**< 0.001**
Duration IV antibiotics	3.7 (2.2)	5.1 (2.8)	**0.007**
Duration oral antibiotics	6.1 (2.7)	8.4 (4.2)	**< 0.001**
Antibiotic timing (number of days from admission to initiation), mean (SD)	0.1 (0.6)	0.1 (0.3)	0.824
Adverse events, *n* (%)	4/123 (3.3)^a^	1/25 (4.0)^b^	0.852
Change of medication due to AE, *n* (%)	3/4 (75.0)^c^	0/1 (0.0)	0.272
Surgery performed, *n* (%)	10/123 (8.1)	14/25 (56.0)	**< 0.001**
Surgical complications, *n* (%)	0/10 (0.0)	0/14 (0.0)	-
ID consult performed, *n* (%)	11/123 (8.9)	4/25 (16.0)	0.290
Intensive care unit admission, *n* (%)	0/123 (0.0)	0/25 (0.0)	-
Representation within 30 days, *n* (%)	5/123 (4.1)	1/25 (4.0)	0.988

^a^ Vomiting/did not tolerate (clindamycin) (2 patients); did not tolerate (augmentin) (1 patient); rash (cephalexin) (1 patient)

^b^ Rash (augmentin)

^c^ Vomiting/did not tolerate (clindamycin) (2 patients); did not tolerate (augmentin) (1 patient)

The children in the uncomplicated disease group were predominantly managed on narrow-spectrum beta-lactam therapy. The primary intravenous (IV) antibiotics used were flucloxacillin or cephazolin (79.3%) with subsequent IV-to-oral transition to flucloxacillin or cefalexin (63.4%) (Table 4). A considerable number of children received IV third-generation cephalosporins (63.6%) in the acute phase.

**Table 4:** Details of antibiotics administered to children with acute bacterial lymphadenitis, stratified by severity

	**Uncomplicated disease**	**Complicated disease**	*p*
	*n* = 123	*n* = 25	
IV antibiotics, *n* (%)			
Anti-Staphylococcal beta-lactam (flucloxacillin or cephazolin)	96/121 (79.3)	18/25 (72.0)	0.423
Third-generation cephalosporin (cefotaxime or ceftriaxone)	77/121 (63.6)	17/25 (68.0)	0.681
Clindamycin	28/121 (23.1)	13/25 (52.0)	**0.003**
Other	19/121 (15.7)	7/25 (28.0)	0.145
PO antibiotics, *n* (%)			
Anti-Staphylococcal beta-lactam (flucloxacillin or cefalexin)	71/112 (63.4)	9/25 (36.0)	**0.012**
Clindamycin	3/112 (2.7)	2/25 (8.0)	0.202
Amoxicillin/clavulanic acid	31/112 (27.7)	12/25 (48.0)	**0.048**
Other	13/112 (11.6)	3/25 (12.0)	0.956

Compared to the uncomplicated disease group, patients with complicated disease were more likely to be given IV clindamycin (52.0% versus 23.1%, *p* = 0.003) (Table 4), mostly as adjunctive therapy alongside a beta-lactam. Oral amoxicillin/clavulanic acid was used significantly more frequently (48.0% versus 27.7%, *p* = 0.048) and oral flucloxacillin or cefalexin less frequently (36.0% versus 63.4%, *p* = 0.012) in the complicated group, though the rationale for this is unclear. Oral clindamycin was given infrequently in both groups (8.0% versus 2.7%, *p* = 0.202).

Although analysis is limited by a small number of culture-positive cases, there was no clear pattern in clindamycin use based on pathogen identified (Supplementary Table 1). Clindamycin was at least as likely to be used in MSSA-associated disease as in Group A Streptococcus-associated disease; in fact, amongst uncomplicated culture-positive cases, clindamycin was used more often in MSSA-related infection (37.5%) than in cases with identified Group A Streptococcus (9.1%).

Adverse events occurred rarely in both the complicated and uncomplicated groups. In only three cases (2.0% overall) was a change of therapy required due to antibiotic-related adverse effects, in all three cases due to oral refusal or vomiting. Oral clindamycin was implicated in two of these cases and oral amoxicillin/clavulanic acid in one case.

## Discussion

Here, we have shown that acute bacterial lymphadenitis predominantly affected previously well preschool-aged children and infants. Most children presented with fever and systemic symptoms in addition to localised swelling. Cervical lymphadenopathy was most common and was typically unilateral. The burden of disease was high in the acute period with hospitalisation of 3–5 days for IV antibiotic therapy and one in six children requiring surgical intervention. Children with complicated bacterial lymphadenitis, comprising soft tissue abscesses or associated retro- or para-pharyngeal collections, had a more severe disease course, requiring prolonged hospitalisation and longer durations of antibiotics, as well as markedly higher rates of surgical intervention. Delay to presentation was a key feature of the complicated disease group; hence, encouragement of prompt medical attention is important for all children with lymphadenitis.

Children with Aboriginal and Torres Strait Islander background were significantly over-represented in the complicated disease group, which is indicative of broader health disparities within Australia, especially in relation to invasive bacterial disease [11–14]. Current efforts are focussed on closing the gap to ensure equity in health outcomes for indigenous Australians as a core priority for the Australian health care system [15, 16].

MSSA and Group A Streptococcus were the two most common pathogens causing lymphadenitis in children in this study, on par with expectations in a low-MRSA-prevalence setting such as Australasia or Europe. In such an environment, an empiric antibiotic regimen with bactericidal activity against both MSSA and Group A Streptococcus should provide optimal spectrum and activity to ensure best clinical outcomes in a great majority of cases. While Group A Streptococcus possesses a multitude of virulence factors and is well known to advance across tissue planes causing severe necrosis, it remains almost universally susceptible to penicillin [17]. As a result, use of a narrow-spectrum anti-Staphylococcal/Streptococcal beta-lactam such as flucloxacillin or cephazolin—as recommended in local guidelines [9]—provides coverage for both Group A Streptococcus and MSSA with superior activity compared to higher-generation cephalosporins or alternative agents such as cotrimoxazole or metronidazole in most contexts.

Nonetheless, a high degree of variability in prescribing practices is apparent in this cohort, as has been seen elsewhere [18]. Of note, the frequent use of third-generation cephalosporins in this dataset is surprising, given their inferior efficacy. Infrequent consultation of infectious disease services may be contributory to this finding. Given rising antimicrobial resistance rates globally [19]—in large part, attributable to the selection pression of excessive antibiotic use including third-generation cephalosporins [20]—the use of such broad spectrum agents should be closely regulated. This is particularly relevant in contexts where narrow spectrum agents such as flucloxacillin are preferable, such as for bacterial lymphadenitis. Close multi-disciplinary engagement moving forward will be important to ensure appropriate use of narrow-spectrum antimicrobials in the context of bacterial lymphadenitis.

Our data demonstrate that uncomplicated disease can be effectively treated with narrow-spectrum beta-lactam therapy alone in most cases; we suggest that clindamycin in complicated disease is not routinely required and should be considered on a case-by-case basis. For complicated disease, clindamycin was used commonly in this case series though evidence for its efficacy in this context is lacking, and poor tolerability of the oral formulation was noted. The use of clindamycin for skin and soft tissue infections is generally predicated on its activity against MRSA, as well as its anti-toxin effects, excellent penetration into soft tissues, and efficacy in killing bacteria in the stationary phase of growth [21, 22]. Resistance rates of Group A Streptococci to clindamycin vary widely from 4 to 40% [17] and are on the increase [23]; hence, this agent is rarely used as monotherapy without knowledge of local antibiograms. The use of clindamycin alongside an anti-Staphylococcal backbone agent for complex *S. aureus* infections with associated bacteraemia has been evaluated via a pilot multi-centred randomised controlled trial in adults and children [24] and has been incorporated as a key arm of an ongoing adaptive platform trial (*S. aureus* Network Adaptive Platform (SNAP) trial) [25]. Its role in invasive Group A Streptococcal infection is well established in children [26] and has been shown to reduce mortality in adult cohorts when used alongside beta-lactams [27, 28].

To date, no randomised controlled trials have compared the use of beta-lactams with and without clindamycin for complicated or uncomplicated acute bacterial lymphadenitis or (more broadly) skin and soft tissue infections in adults or children. Equipoise is easy to justify in this context, and only in the context of a prospective randomised controlled trial can the impact of adjunctive clindamycin on outcomes in acute bacterial lymphadenitis be ascertained. Such studies are welcomed to provide robust data to guide choice of antibiotic therapy for children with acute bacterial lymphadenitis, particularly complicated disease.

Our study reveals that children with acute bacterial lymphadenitis bear a significant burden in terms of IV antibiotic duration and hospitalisation with 3.7 ± 2.2 days of IV therapy given in uncomplicated disease and 5.1 ± 2.8 days in complicated disease. Children in our study with uncomplicated disease received a total of 9.7 ± 3.4 days of antibiotic therapy (increasing to 13.5 ± 5.8 days in the complicated disease group), likely reflecting our hospitalised cohort skewed towards the severe end of the disease spectrum. Shorter durations may be sufficient, particularly for outpatients with milder disease, and length of therapy should always be guided by clinical response. The optimal duration of therapy for bacterial lymphadenitis is untested in a randomised controlled trial setting, and most experts agree that 7 days is sufficient in most cases. Expert guidance (based on observational data) varies but in general, a minimum of 2–3 days of IV antibiotics are recommended in patients with systemic features (such as our hospitalised cohort), and oral stepdown can occur after adequate clinical response [29]. Given the risks associated with prolonged antibiotic use—including emergent antimicrobial resistance, hospital-acquired infections, and family stressors arising from hospitalisation—further data is urgently needed on optimised duration of therapy [29]. Where clinical response is favourable, early oral stepdown or transition to a hospital-in-the-home service should be considered.

This study has several limitations and must be interpreted in that context. Of note, this cohort included only patients presenting to hospital and, hence, did not incorporate the milder end of the disease spectrum, which is managed predominantly in the outpatient setting. As a single-centre retrospective study, the current dataset captures local demographics (from both host and pathogen points of view) as well as prescribing patterns. The Australian context is likely generalisable to other low-MRSA prevalence settings across Europe, East Asia, and Australasia; however, close analysis of local antibiograms is always recommended.

This study supports the use of flucloxacillin or a narrow-spectrum first-generation cephalosporin in most cases of uncomplicated lymphadenitis, with close clinical monitoring. Early imaging is recommended to identify complicated cases of lymphadenitis with associated abscesses or soft tissue collections, which would warrant prompt surgical intervention. In such cases, consultation with an infectious diseases expert is suggested to guide appropriate antibiotic choices, taking into account patient-specific factors, culture results, and local antibiogram information. The role of adjunctive clindamycin in complicated disease is unclear and warrants further investigation, as does the optimal duration of therapy, balancing the need for efficacious treatment with the risks of prolonged IV antibiotics and hospitalisation.

## Supplementary Information

Below is the link to the electronic supplementary material.

Supplementary file1 (DOCX 17 kb)

## Data Availability

The data that support the findings of this study are available from the corresponding author upon reasonable request.

## Abbreviations

IV: Intravenous
MRSA: Methicillin-resistant *Staphylococcus aureus*
MSSA: Methicillin-susceptible *Staphylococcus aureus*
USA: United States
