# Supplementary material for: Acute bacterial lymphadenitis in children: a retrospective, cross-sectional study
Source: Eur J Pediatr. 2023 Mar 7;182(5):2325–33. doi: 10.1007/s00431-023-04861-0 (PMC10175353; doi:10.1007/s00431-023-04861-0)
Supplement: Supplementary file 1 — Supplementary file1 (DOCX 17 kb) [file 431_2023_4861_MOESM1_ESM.docx]

**Acute bacterial lymphadenitis in children: a retrospective, cross-sectional study**

**Supplementary Table 1. Summary of antibiotics administered for culture-positive cases of lymphadenitis**

|  | **All** n=35 | | | | | **Uncomplicated Disease** n=22 | | | | | **Complicated Disease** n=13 | | | | |
| --- | --- | --- | --- | --- | --- | --- | --- | --- | --- | --- | --- | --- | --- | --- | --- |
|  | Group A Streptococcus | Group B Streptococcus | Methicillin-susceptible *Staphylococcus aureus* | Methicillin-resistant *Staphylococcus aureus* | Other | Group A Streptococcus | Group B Streptococcus | Methicillin-susceptible *Staphylococcus aureus* | Methicillin-resistant *Staphylococcus aureus* | Other | Group A Streptococcus | Group B Streptococcus | Methicillin-susceptible *Staphylococcus aureus* | Methicillin-resistant *Staphylococcus aureus* | Other |
| n | 15 | 1 | 17 | 2 | 0 | 11 | 1 | 8 | 2 | 0 | 4 | 0 | 9 | 0 | 0 |
| IV antibiotics | flucloxacillin (10); cefotaxime/ceftriaxone (10); clindamycin (4); benzylpenicillin (6); piperacillin/tazobactam (1); tobramycin (1) | benzylpenicillin; ampicillin; cefotaxime; gentamicin (1) | flucloxacillin (16); cefotaxime/ceftriaxone (10); clindamycin (8); benzylpenicillin (1) | flucloxacillin (2); cefotaxime/ceftriaxone (1); clindamycin (1) |  | flucloxacillin (8); cefotaxime/ceftriaxone (6); benzylpenicillin (4); clindamycin (1); piperacillin/tazobactam (1); tobramycin (1) | benzylpenicillin; ampicillin; cefotaxime; gentamicin (1) | flucloxacillin (8); cefotaxime/ceftriaxone (6); clindamycin (3); benzylpenicillin (1) | flucloxacillin (2); cefotaxime/ceftriaxone (1); clindamycin (1) | - | cefotaxime/ceftriaxone (4); clindamycin (3); flucloxacillin (2); benzylpenicillin (2) | - | flucloxacillin (8); clindamycin (5); cefotaxime/ceftriaxone (4) | - |  |
| PO antibiotics | amoxicillin (5); cefalexin (4); augmentin (3); flucloxacillin (1); penicillin V (1); nil (1) | nil | flucloxacillin (4); cefalexin (9); clindamycin (1); cotrimoxazole (2); augmentin (1); erythromycin (1) | flucloxacillin (1); augmentin (1); cefalexin (1) |  | cefalexin (4); amoxicillin (3); flucloxacillin (1); augmentin (1); penicillin V (1); nil (1) | nil | flucloxacillin (1); cefalexin (4); cotrimoxazole (2); augmentin (1) | flucloxacillin (1); augmentin (1); cefalexin (1) | - | amoxicillin (2); augmentin (2) | - | cefalexin (5); flucloxacillin (3); clindamycin (1); erythromycin (1) | - |  |
| Patients receiving clindamycin (IV or PO), n (%) | 4 (26.7) | 0 (0.0) | 8 (47.1) | 1 (50.0) |  | 1 (9.1) | 0 (0.0) | 3 (37.5) | 1 (50.0) | - | 3 (75.0) | - | 5 (55.6) | - |  |

**Supplementary Table 2. Antibiotic usage and surgical interventions implemented for all culture-positive cases.**

| Organism isolated | Group A Streptococcus n = 15 | Group B Streptococcus n = 1 | Methicillin-susceptible *Staphylococcus aureus* n = 17 | Methicillin-resistant *Staphylococcus aureus* n = 2 |
| --- | --- | --- | --- | --- |
| Surgery required, n (%) | 7/15 (46.7) | 0/1 (0.0) | 11/17 (64.7) | 0/2 (0.0) |
| Duration intravenous antibiotics (days), mean (SD) | 3.9 (2.1) | 15 | 6.0 (2.7) | 5.5 (0.7) |
| Duration oral antibiotics (days), mean (SD) | 7.1 (3.9) | 0 * | 7.3 (4.4) | 8.5 (2.1) |
| Total antibiotic duration (days), mean (SD) | 10.9 (5.3) | 15 | 13.3 (6.0) | 14.0 (1.4) |

* Note this infection occurred in a neonate hence entire antibiotic duration given intravenously.
